# Editorial: Reviews in sleep: 2024

**DOI:** 10.3389/frsle.2026.1926567

**Published:** 2026-07-29

**Authors:** Ritchie Edward Brown, Dalva L. R. Poyares, Stephen Sheldon, Radhika Basheer

**Affiliations:** 1 Laboratory of Neuroscience, Veterans Administration Boston Healthcare System and Harvard Medical School, Department of Psychiatry, West Roxbury, MA, United States; 2 Departamento de Psicobiologia, Universidade Federal de São Paulo, São Paulo, Brazil and Sleep Institute, São Paulo, Brazil; 3 Emeritus, Pediatrics (Pulmonary and Sleep Medicine), Northwestern University, Feinberg School of Medicine, Chicago, IL, United States; 4 Emeritus, Sleep Medicine Center, Ann and Robert H. Lurie Children's Hospital of Chicago, Chicago, IL, United States

**Keywords:** circadian, infant, non-pharmacological, oxytocin, pediatric, sleep fragmentation, sleep quality, stress

We are pleased to introduce this Research Topic entitled “*Reviews in sleep: 2024*.” These eight review articles encompass the broad range of sleep and circadian research with foci on pediatric sleep, non-pharmacological treatments to improve sleep quality and novel neurobiological substrates which may mediate the effects of physical and social stressors on sleep-wake physiology.

Infancy is a time of life when most animals, including humans, spend the majority of their time sleeping (Roffwarg et al., 1966). Animal studies have elucidated some of the brain mechanisms which regulate the development of sleep physiology (Gvilia et al., 2017). However, less attention has been devoted to the analysis of sleep in infant humans, and to its dysregulation, despite the considerable clinical implications (Sheldon and Owens, 2024). Three of the eight articles in this Research Topic focus on this Research Topic. Gilchrist et al. describe normative data on the maturation and consolidation of infant sleep-wake during the first 6 months of life using a scoping review from 2000 to 2024, integrating and comparing findings from studies using the two most commonly used methodologies, actigraphy, and sleep diaries. Byeon et al. address the vital and understudied topic of sleep fragmentation in critically ill children in the pediatric care unit. Sleep in such a setting is often disrupted by environmental stimuli, sedation and clinical measurements or treatments, resulting in loss of deep restorative sleep. Importantly, they conclude that emerging evidence suggests links between fragmented sleep and altered neurodevelopmental trajectories and/or increased risk of pediatric post-intensive care syndrome involving disturbed cognition and delirium. The authors propose that changes in lighting, auditory disturbances and other environmental factors may prove beneficial in reducing sleep disruption and improving clinical outcomes, as in adult ICUs. The final of these three developmentally-oriented studies focuses on pediatric narcolepsy. Narcolepsy is usually diagnosed in the late teen years or early twenties. However, symptoms of narcolepsy can appear earlier, and there is often a long delay between the appearance of symptoms and the diagnosis. Here, Liu et al. describe a case report of a 4-year-old girl with narcolepsy and use information from this case to suggest a comprehensive treatment approach for pediatric narcolepsy patients combining medication and behavioral approaches.

Adequate and appropriately timed sleep is one of the three pillars of health (Lim et al., 2023), together with good nutrition and exercise. The sleep and circadian research field has some of the most impressive examples of translating basic neuroscience findings into elucidating the pathophysiology of disease and developing novel treatments to improve sleep. For instance, the discovery of the orexin/hypocretin neurotransmitter system led to the discovery that loss of orexin/hypocretin neurons is the cause of most cases of narcolepsy and to the development of a novel class of sleep-promoting drugs, the dual orexin receptor antagonists (DORAs) (Coleman et al., 2017). Similarly, the discovery of clock genes and proteins and their transcriptional/translational feedback loop led to the Nobel Prize and to the discovery that mutations in clock genes can lead to circadian rhythm disorders such as advanced or delayed sleep phase disorder (Lane et al., 2023). Accordingly, there is considerable interest in leveraging new basic neuroscience findings in the sleep and circadian field to develop novel behavioral, pharmacological, and brain stimulation techniques to treat a wide variety of brain and body disorders (Brown et al., 2022). The five remaining articles in this Research Topic discuss understudied non-pharmacological treatments to improve sleep or circadian rhythms, as well as novel neurobiological targets which might be targeted to ameliorate the negative effects of physical or social stressors on sleep-wake physiology. The comprehensive review article by Morin et al., provides an up-to-date primer on the neurobiology of circadian rhythms for researchers and clinicians and a perspective on their implications for the development of novel “chrono-interventions” to optimize health outcomes by aligning physiological processes and medical treatments with the body's natural rhythms. Other articles in this Research Topic focus on different types of approaches to improve sleep quality and treat common sleep disorders. Steinmane and Fernate review studies using a relatively unexplored, non-pharmacological strategy to improve sleep quality, namely breathing exercises, including studies involving deep breathing, diaphragmatic breathing, mindful breathing, respiratory muscle training and pursed lip breathing. They find that most studies using these techniques for 4 weeks or longer resulted in improved sleep quality, typically assessed using the Pittsburgh sleep quality index. They propose that breathing exercises may improve sleep through modulation of autonomic nervous system function and stress reduction.

A brain region involved in integrating autonomic regulation and stress responses as well as sleep-wake control is the paraventricular nucleus of the hypothalamus. Sun et al. review diverse cellular mechanisms in the PVN such as neuronal plasticity, neuroinflammation and oxidative stress which may collectively drive sustained sympathetic overaction and HPA axis dysregulation in obstructive sleep apnea. Another brain system involved in stress-mediated changes in sleep is the brain oxytocin system. Boccalaro et al. review and discuss preclinical and human studies which suggest that oxytocin release may contribute to sleep stability and stress reduction through modulation of hippocampal, amygdala and thalamocortical circuits. Conversely, social isolation, a strong stressor, reduces oxytocin signaling and disrupts sleep-wake dynamics. The availability of intra-nasal oxytocin for human use suggests the possibility of clinical trials to improve insomnia. Finally, Zhang et al. evaluated the efficacy and safety of non-pharmacological therapies for primary insomnia such as acupuncture, acupressure and cognitive behavioral therapy (CBT) through a network meta-analysis, with the goal to provide evidence-based guidance for clinicians. All seven non-pharmacological therapies tested were effective in improving various outcome measures for primary insomnia with a low to moderate certainty of evidence. CBT was particularly effective in reducing sleep latency and improving sleep efficiency, whereas acupuncture was effective in prolonging total sleep time. Overall, non-pharmacological interventions were superior to pharmacological interventions for improving sleep parameters in patients with primary insomnia.

We hope the readers find these articles of interest and encourage further submissions on unique mechanistic aspects of sleep and circadian physiology/pathophysiology and their treatment.
